# Right Inferior Parietal Lobule Activity Is Associated With Handwriting Spontaneous Tempo

**DOI:** 10.3389/fnins.2021.656856

**Published:** 2021-06-09

**Authors:** Laura Bonzano, Ambra Bisio, Ludovico Pedullà, Giampaolo Brichetto, Marco Bove

**Affiliations:** ^1^Department of Neuroscience, Rehabilitation, Ophthalmology, Genetics, Maternal and Child Health, University of Genoa, Genoa, Italy; ^2^Section of Human Physiology, Department of Experimental Medicine, University of Genoa, Genoa, Italy; ^3^Scientific Research Area, Italian Multiple Sclerosis Foundation, Genoa, Italy; ^4^Rehabilitation Center, Italian Multiple Sclerosis Society, Genoa, Italy; ^5^Ospedale Policlinico San Martino, Istituto di Ricovero e Cura a Carattere Scientifico, Genoa, Italy

**Keywords:** handwriting, inferior parietal lobule, spontaneous tempo, writing center, functional magnetic resonance imaging

## Abstract

Handwriting is a complex activity including motor planning and visuomotor integration and referring to some brain areas identified as “writing centers.” Although temporal features of handwriting are as important as spatial ones, to our knowledge, there is no evidence of the description of specific brain areas associated with handwriting tempo. People with multiple sclerosis (PwMS) show handwriting impairments that are mainly referred to as the temporal features of the task. The aim of this work was to assess differences in the brain activation pattern elicited by handwriting between PwMS and healthy controls (HC), with the final goal of identifying possible areas specific for handwriting tempo. Subjects were asked to write a sentence at their spontaneous speed. PwMS differed only in temporal handwriting features from HC and showed reduced activation with a subset of the clusters observed in HC. Spearman’s correlation analysis was performed between handwriting temporal parameters and the activity in the brain areas resulting from the contrast analysis, HC > PwMS. We found that the right inferior parietal lobule (IPL) negatively correlated with the duration of the sentence, indicating that the higher the right IPL activity, the faster the handwriting performance. We propose that the right IPL might be considered a “writing tempo center.”

## Introduction

Handwriting is one of the most common daily activities performed by adults in a variety of settings (Dixon et al., 1993). Also, it is one of the most important tools of communication and is a uniquely human skill. Handwriting is a complex activity requiring cognitive, kinesthetic, and perceptual-motor components, including motor planning and visuomotor integration (Tseng and Cermak, 1993). In the literature, two brain areas have been proposed to be uniquely associated with writing and for this reason named “writing centers”: Exner’s area, i.e., a region of the dorsal premotor cortex associated with the writing activity (Yuan and Brown, 2015), and the visual word form area, i.e., a region of the left ventral temporal lobe medial to the fusiform gyrus associated with the visual perception of words compared with non-word forms (Cohen et al., 2000; Dehaene et al., 2010; Nakamura et al., 2012; Wilson et al., 2013). Furthermore, a region located around the left intraparietal sulcus, from the anterior part of the left superior parietal lobule (SPL) to the upper part of the left supramarginal gyrus, was found to be crucial to generate a sequential movement for writing and described as “parietal writing center” (Kinsbourne and Rosenfield, 1974; Basso et al., 1978; Auerbach and Alexander, 1981; Takayama et al., 1994; Katanoda et al., 2001; Menon and Desmond, 2001; Sugihara et al., 2006; Roux et al., 2010; Rapp and Dufor, 2011). Although temporal features of handwriting are important as spatial ones, to our knowledge, there is no evidence of the description of specific areas associated with handwriting tempo.

In general, each individual has its own spontaneous and preferred rhythm in performing a task. In literature, it has been suggested that individual spontaneous movement tempo (SMT) refers to the rate of a putative endogenous oscillator (Bisio et al., 2015). Further, behavioral measures demonstrated that SMT and preferred perceptual tempo are strongly correlated, indicating this oscillator as a central mechanism (McAuley et al., 2006; Michaelis et al., 2014). As a consequence, SMT would not be merely confined to the motor domain, but it would be the expression of an overall mechanism, which could influence the perception of time.

An essential contribution to handwriting skills comes from those brain areas processing visual information. In fact, vision plays a major role in the temporal control of handwriting (van Doorn and Keuss, 1992); it is commonly recognized that vision is involved in the identification of objects in our visual environment (“what”) and the place where we locate those objects (“where,” or more recently called “vision-for-action”) (Mishkin et al., 1983; Goodale et al., 2004). However, an equally important ability is how we compute the time in which (“when”) visual events occur (Battelli et al., 2007). All these functions are related to different neural pathways, originating from the primary visual cortex, in which associative areas integrating visual inputs and located in the parietal or temporal lobes are present.

In general, visual input processing is crucial in cognitive skills dealing with the domains of attention, executive functions, memory, and visuospatial abilities. Cognitive impairment affects a large part of people with multiple sclerosis (PwMS). Recently, it has been shown that rehabilitation treatments of attention, information processing, and executive functions in PwMS may be affected through enhanced recruitment of brain networks mainly located in the parietal associative areas and referring to visual input processing (Filippi et al., 2012; Bonzano et al., 2020). In this context, it should be considered that functional decline among PwMS embraces worsening of handwriting (Rosenblum and Tamar Weiss, 2010). Indeed, handwriting deficits are common in multiple sclerosis, but studies on this aspect of the disease are rare (Wellingham-Jones, 1991; Schenk et al., 2000; Bisio et al., 2017). In particular, PwMS show handwriting impairments that are mainly referred to the temporal features of the task. Schenk et al. (2000) found that, although the script of PwMS, compared with healthy subjects, was still legible, the writing speed decreased and stroke duration increased (Schenk et al., 2000). In line with this study, we recently showed that handwriting movements of PwMS significantly differed from those of healthy controls (HC) in the time spent to write a sentence, which was due to increased duration of the words and the spacing between words (Bisio et al., 2017).

The aim of the present work was to quantitatively characterize the handwriting movement in PwMS and HC and to investigate the associated brain activation patterns by means of a magnetic resonance (MR)-compatible tablet during functional MR imaging (fMRI), with the final goal of identifying possible writing centers specific for handwriting tempo.

Specifically, following all these findings and taking into account that handwriting refers to sensorimotor processes related to visual guidance of hand movement resulting in the formation of visual shapes associated with words (Yuan and Brown, 2015), we hypothesized that brain areas related to handwriting tempo are located in the associative parietal areas processing visual information and more likely in those areas able to link action to time perception and attention.

## Materials and Methods

### Subjects

PwMS in a stable phase of the disease (i.e., no relapses or worsening in the previous 3 months) reporting handwriting impairments during a preliminary brief interview were recruited for this study. Inclusion criteria were as follows: age more than 18 years; both sexes; impairment in handwriting; and mild and moderate muscle strength deficit in the upper limb as assessed by the Medical Research Council scale (Compston, 2010) (muscle strength with grade 4 in all muscle groups or grade 3 in no more than 2 joints). Exclusion criteria were as follows: Mini Mental State Examination (Folstein et al., 1975) less than 26; Modified Ashworth scale to evaluate muscle tone of the upper limb (Bohannon and Smith, 1987) more than 3 in at least two muscle groups; and inability to perform simple handwriting movements. PwMS were also evaluated using the Expanded Disability Status Scale (EDSS) (Kurtzke, 1983).

Eighteen PwMS (12 females, age = 45.1 ± 11.0 years, disease duration = 15.6 ± 10.1 years) were involved in this study. Fourteen subjects had a relapsing–remitting disease course, and four had a secondary progressive disease course; median EDSS was 4 (Supplementary Table 1).

A group of 18 age- and sex-matched HC (12 females, age = 40.1 ± 11.4 years) was included for comparisons. All the included subjects were naïve to the specific purpose of the study and right-handed according to the Edinburgh Handedness Inventory (Oldfield, 1971). Informed consent was obtained according to a procedure approved by the local ethics committee (Comitato Etico Regionale Liguria, IRCCS Azienda Ospedaliera Universitaria San Martino–IST, Genoa, Italy; P.R. 258REG2015) and according to the Declaration of Helsinki.

### Behavioral Data Acquisition and Analysis

The MR-compatible touch-sensitive tablet SMART TAB (E.M.S., S.r.l., Bologna, Italy) was used to acquire handwriting movements, as previously reported (Bisio et al., 2016). From the recorded traces, the kinematic parameters describing handwriting movements were computed by means of a custom-made Matlab software (MathWorks, Portola Valley, CA, United States); more details can be found elsewhere (Bisio et al., 2017). The experimental setup is represented in Supplementary Figure 1A.

Handwriting performance was assessed considering the sentence as a whole, and the words and the spacing between words separately. In particular, in order to provide a spatiotemporal description of the subjects’ performance, we considered the following parameters: the duration (i.e., the time employed by the subject to write), the length and the height of the sentence; the duration and the length of the words (considering the sum of the words); and the duration and the length of the spacing between words (considering the sum of the two intervals between words) (Supplementary Figure 1B).

### MRI Acquisition

MRI examination was performed on a 1.5-Tesla MR system (Signa Excite HDxt, General Electric Healthcare, Milwaukee, WI, United States) and included the following series acquired in the transverse plane and covering the whole brain: fluid-attenuated inversion recovery (FLAIR) sequence [slice thickness = 5 mm; repetition time (TR) = 9,002 ms; echo time (TE) = 97.5 ms; inversion time = 2,250 ms; flip angle = 90°; field of view (FOV) = 240 × 240 mm; matrix = 512 × 512] to exclude incidental findings in the HC; T2-weighted sequence (slice thickness = 4.5 mm; gap = 0.5 mm; *TR* = 6,300 ms; *TE* = 123.7 ms; FOV = 260 × 260 mm; matrix = 256 × 256) was used as structural reference for the fMRI acquisition; T2^∗^-weighted single-shot echo-planar imaging (EPI) sequences (slice thickness = 4.5 mm; gap = 0.5 mm; *TR* = 3,000 ms; *TE* = 40 ms; FOV = 260 × 260 mm; matrix = 64 × 64) for fMRI. The functional scan included 63 brain volumes, and the first three volumes (i.e., 9 s) were discarded because of non-steady magnetization.

### Functional MRI Procedure

During fMRI, every subject had to perform the handwriting motor task (i.e., active condition) or prompted to stay still (i.e., rest condition), according to a block-designed paradigm consisting of three 30-s rest periods alternating with three 30-s active periods. Subjects were instructed to maintain their eyes open for the whole duration of the fMRI sessions, looking at a fixation cross during the rest periods, in order to maintain visual inputs and avoid potential artifactual activations.

Throughout the handwriting motor task period, subjects had to write at their spontaneous tempo the Italian sentence “Il sole scalda” (i.e., “The sun warms”), in cursive font more times on subsequent lines when a “go” signal was provided until a “stop” signal. This sentence was chosen because it consists of simple words very common in Italian language and not evoking high cognitive demand and/or specific emotions. A plastic-made stylus without ink was used to write on the surface of an innovative MR-compatible touch-sensitive tablet, and a black line reproducing the written trace appeared on a screen to allow real-time monitoring of what subjects were writing, by means of an *ad hoc*-developed software tool (Bisio et al., 2016).

### Functional MRI Data Preprocessing

SPM12 software (Wellcome Department of Imaging Neuroscience, London, United Kingdom) was used for fMRI processing (Friston et al., 1995). For each participant, the first image was used as a reference to which all the subsequent scans were realigned, and the six parameters describing the rigid body transformation between each source image and the reference image were used to re-sample each image to apply motion correction. Then, slice timing was applied to minimize timing errors between slices, and the functional images were normalized to the Montreal Neurological Institute (MNI) template brain image using a 12-parameter affine transformation (resampled voxel size = 2 mm isotropic) and smoothed with an 8-mm full-width at half-maximum isotropic Gaussian kernel to increase the signal-to-noise ratio.

### Statistical Analysis

#### Behavioral Parameters

The statistical analysis of the handwriting parameters was performed by means of SPSS Statistics 20 (IBM, Endicott, NY, United States). Normality was checked by means of the Shapiro–Wilk tests. Independent *t*-tests were applied in case of normally distributed parameters, while the Mann–Whitney tests were used in case of non-normally distributed data. Sentence length and height, and word length followed a normal distribution, while sentence duration, word duration, the duration and length of the spacing between words did not. The presence of outliers was checked considering mean values ± 2 ^∗^ standard deviation in case of normally distributed data, and (inferior quartile −1.5^∗^interquartile interval, superior quartile + 1.5 ^∗^ interquartile interval) in case of non-normally distributed data. No HC and PwMS duration values were outside these intervals, and thus no outliers were present in our dataset (Tukey, 1977).

#### Functional MRI Data

After pre-processing of fMRI data, a general linear model was used to identify the voxels with task-related signal changes at the individual level. Task-related *t* contrast images were created for each subject and then introduced into a second-level random-effect analysis to allow for population inferences. The corresponding group activation maps were determined for PwMS and HC using one-sample *t*-tests with a height threshold of *p* < 0.05 family-wise error (FWE) corrected and a minimum cluster size arbitrarily set to 20 voxels. Statistical comparisons between groups were performed with two-sample *t*-tests (PwMS > HC and HC > PwMS), with a height threshold of *p* < 0.001 and a minimum cluster size arbitrarily set to 20 voxels. The first eigenvariate of the blood oxygenation level-dependent (BOLD) signal was extracted for the activation clusters, which resulted in statistical significance from this contrast analysis.

Pearson’s or Spearman’s correlation analyses were applied as appropriate to assess the relationship between the kinematic parameters resulting in significant difference between PwMS and HC and the first eigenvariate of the BOLD signal in the activation clusters resulting in statistical significance from the contrast analysis (PwMS vs. HC). The Bonferroni correction for multiple testing was applied (*p* = 0.05/8 = 0.0063).

## Results

### Behavioral Data

**FIGURE 1:**
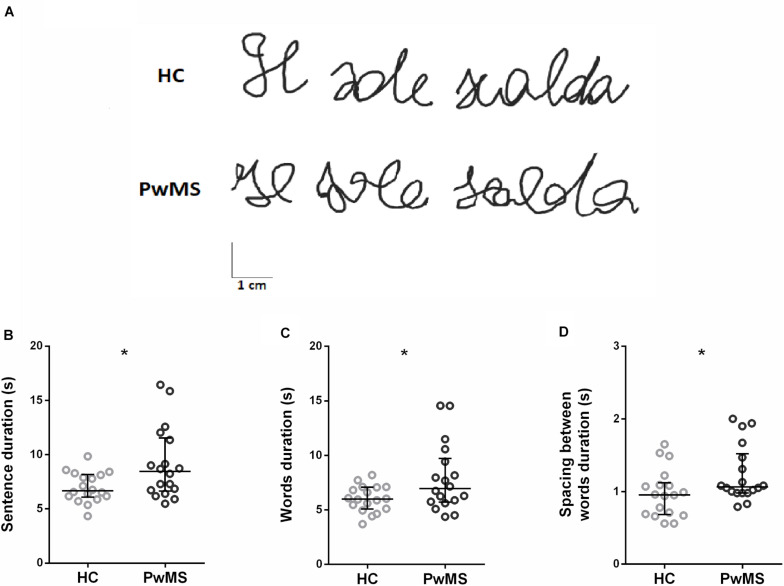
**(A)** Examples of written traces by two representative participants, one for healthy control (HC) group and one for people with multiple sclerosis (PwMS) group. **(B–D)** Kinematic parameters describing the handwriting task found to be significantly different between HC (gray) and PwMS (black): **(B)** duration of the sentence, **(C)** duration of the words, and **(D)** duration of the spacing between words. Each circle represents the average value for a single subject. The horizontal line indicates the group median value, and the error bars show the interquartile interval *p < 0.05.

Figure 1A shows an example of the sentence written by one participant from HC and one from PwMS group. Concerning data analysis, during the handwriting task, PwMS were significantly slower than HC, as indicated by the significant increase of the duration of the sentence (*Z* = −2.56, *p* = 0.01; Figure 1B) and of its components, i.e., the words (*Z* = −2.06, *p* = 0.04; Figure 1C) and the spacing between words (*Z* = −2.21, *p* = 0.03; Figure 1D). No significant differences between groups were found in the other kinematic parameters. A detailed description of the result of the kinematic analysis is reported in Table 1.

**TABLE 1:** Kinematic parameters describing the handwriting task performed by PwMS and HC.

**Parameter**	**PwMS group**	**HC group**	**Statistics**
Sentence duration (s)	8.46 [6.72, 11.36]	6.66 [6.16, 7.88]	*z* = −2.56, *p* = 0.01*
Sentence length (mm)	121.74 ± 6.39	138.19 ± 7.32	*t* = 1.69, *p* = 0.10
Sentence height (mm)	15.92 ± 1.64	19.96 ± 1.55	*t* = 1.79, *p* = 0.08
Word duration (s)	6.94 [5.74, 9.42]	5.99 [5.10, 6.80]	*z* = −2.06, *p* = 0.04*
Words length (mm)	106.42 ± 6.23	119.89 ± 6.51	*t* = 1.49, *p* = 0.14
Spacing between word duration (s)	1.07 [0.98, 1.47]	0.96 [0.69, 1.09]	*z* = −2.21, *p* = 0.03*
Spacing between words length (mm)	13.77 [8.20, 20.76]	14.77 [12.09, 23.87]	*z* = 1.27, *p* = 0.21

Data are reported as mean ± standard deviation when normally distributed and as median [interquartile interval], when not normally distributed. PwMS, people with multiple sclerosis; HC, healthy controls. *Indicates statistical significance.

### Functional MRI Data

**FIGURE 2:**
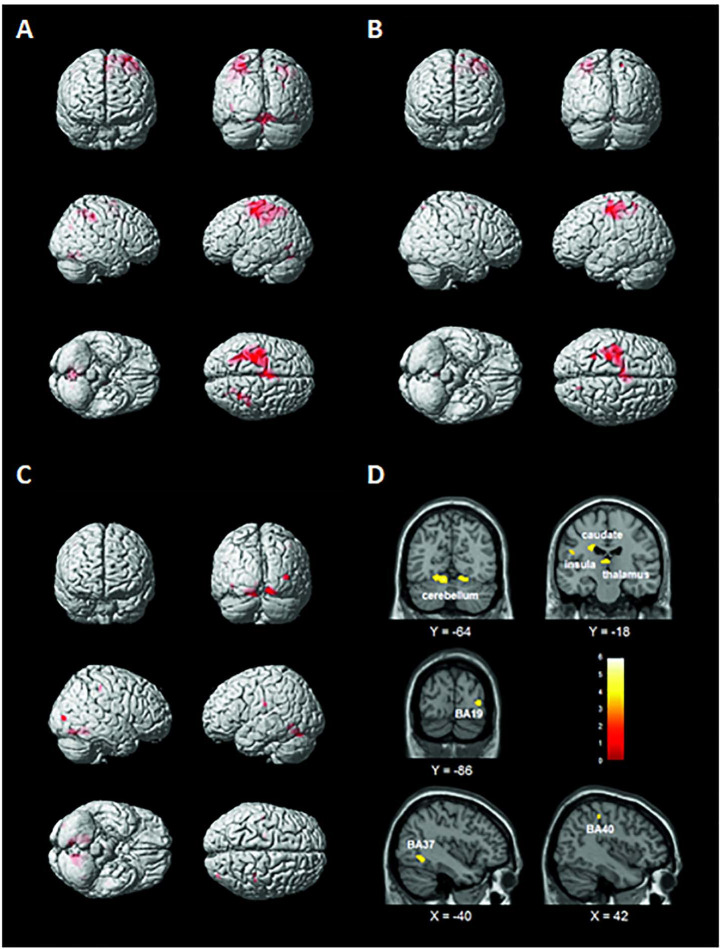
Brain activation patterns elicited by the handwriting task displayed on a rendering surface. **(A)** Healthy controls (HC), **(B)** people with multiple sclerosis (PwMS), and **(C)** statistical contrast between the two groups (HC > PwMS). See Supplementary Table 2. **(D)** Functional MRI (fMRI) sections reporting the clusters of activation found to be significantly more active in the HC than in the PwMS group during the handwriting task (statistical contrast: HC > PwMS). Images are displayed in neurological convention. See Table 2 for details.

Figure 2 and Supplementary Table 2 show the activation patterns found in the two groups while performing the handwriting task. HC significantly activated the left precentral gyrus [Brodmann’s area (BA) 4 and BA6], middle temporal and occipital gyri (BA37), thalamus, left and right inferior parietal lobules (IPLs) (BA40), right precuneus (BA7 and 19), SPL (BA7), inferior temporal gyrus (BA19), and cerebellum. PwMS mainly activated the left precentral gyrus (BA4 and BA6), middle and superior frontal gyri (BA6), and right SPL (BA7) and cerebellum.

**TABLE 2:** Brain regions resulting from the statistical contrasts between the brain activation patterns of the two groups during the handwriting motor task (*p* < 0.001, minimum cluster size *k* = 20 voxels).

**Comparison**	**Cluster size**	**Voxel T**	**Voxel Z**	**MNI coordinate: x y z (mm)**	**Laterality**	**Anatomical location**	**Brodmann’s area**
HC > PwMS	464	6.02	4.93	12 −56 −10	Right	Cerebellum (lobules IV–V)	
		5.06	4.34	16 −70 −14	Right	Cerebellum (lobule VI)	
	371	4.93	4.25	−10 −64 −16	Left	Cerebellum (lobule VI)	
		4.41	3.89	−16 −70 −20	Left	Cerebellum (lobule VI)	
		4.87	4.21	−6 −54 −4	Left	Cerebellum (lobules IV–V)	
	47	3.45	3.17	−14 −14 32	Left	Caudate	
	60	4.14	3.7	−2 −18 12	Left	Thalamus	
		3.71	3.37	4 −26 10	Right	Thalamus	
	47	4.04	3.63	36 −86 6	Right	Middle occipital gyrus	19
	41	3.78	3.43	−40 −54 −10	Left	Fusiform gyrus	37
	28	3.77	3.42	−48 −20 24	Left	Insula	13
	30	3.72	3.39	42 −32 46	Right	Inferior parietal lobule	40
PwMS > HC	*No suprathreshold clusters*						

*MNI, Montreal Neurological Institute; PwMS, people with multiple sclerosis; HC, healthy controls.*

As shown in Figure 2 and Table 2, the statistical contrast between groups revealed that the bilateral cerebellum (lobules IV, V, and VI) and thalamus, left caudate, insula (BA13), fusiform gyrus (BA37), right middle occipital gyrus (BA19), and IPL (BA40) were more active in HC than PwMS (HC > PwMS). No suprathreshold clusters were found in the opposite direction of the t-contrast (PwMS > HC). The first eigenvariate of the BOLD signal was extracted in the regions of interests derived from the significant activation clusters.

### Relationship Between Handwriting Parameters and Functional MRI Data

**FIGURE 3:**
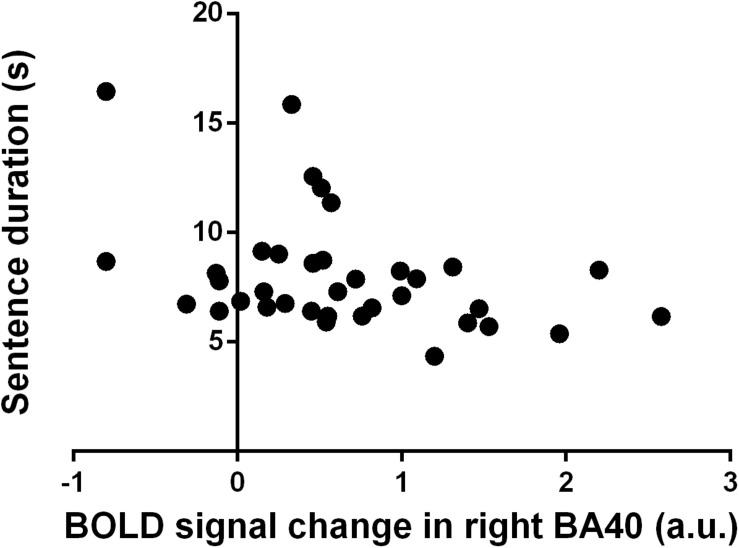
Correlation between blood oxygenation level-dependent (BOLD) signal change in right Brodmann’s area (BA) 40 (a.u.) and sentence duration (s). Each dot refers to a single participant from both healthy controls and people with multiple sclerosis groups.

Spearman’s correlation analysis on the data of the two groups pooled together showed significant relationships between handwriting kinematic parameters and fMRI activity, indicating that the higher the activity, the faster the performance. In detail, BOLD signal in left BA37 and right BA40 negatively correlated with the duration of the sentence (BA37: *r* = −0.35, *p* = 0.02; BA40: *r* = −0.42, *p* = 0.0061) and of the words (BA37: *r* = −0.32, *p* = 0.02; BA40: *r* = −0.34, *p* = 0.02). BOLD signal in right BA19 negatively correlated with the duration of the spacing between words (*r* = −0.29, *p* = 0.04). BOLD signal in the left caudate, left insula, and left and right thalami negatively correlated with the duration of the sentence (respectively, *r* = −0.32, *p* = 0.03; *r* = −0.29, *p* = 0.04; and *r* = −0.30, *p* = 0.04). Furthermore, BOLD signal in the left insula negatively correlated also with word duration (*r* = −0.29, *p* = 0.04). The Bonferroni correction for multiple testing indicated that only the relationship between the right BA40 (IPL) and the duration of execution of the sentence was statistically significant (Figure 3).

## Discussion

In this work, we demonstrated that the activity of the right IPL is related to the duration of execution of a handwriting sentence. Here, we showed that PwMS were slower than HC in the execution of the whole sentence and in both the word writing phase and the writing preparation phase (i.e., spacing between words). During handwriting, PwMS showed a reduced brain activation pattern as compared with HC, mainly including activation clusters located in the left premotor and motor areas and the right SPL and cerebellum. On the other hand, HC significantly activated the left premotor and motor areas and the right cerebellum, the left middle temporal and occipital gyri, and the thalamus. On the right brain hemisphere, the precuneus, the SPL, and the inferior temporal gyrus were active during handwriting. Finally, there was a significant activation in the bilateral IPL. From the contrast analysis, the bilateral cerebellum (lobules IV–VI) and thalamus; the left caudate, insula, and fusiform gyrus; and the right middle occipital gyrus and IPL were found to be more active in HC than PwMS. The first eigenvariate of the BOLD signal in these regions correlated with the handwriting parameters, which resulted in significant difference between PwMS and HC. Although Spearman’s correlation analysis showed different significant correlations between handwriting temporal parameters and the brain activity, only the relationship between the right IPL and the time to execute the handwriting sentence survived the Bonferroni correction. In particular, the right IPL negatively correlated with the duration of the sentence, indicating that the higher the brain area activity, the faster the handwriting performance.

Handwriting brain activations in HC were in line with those reported in the activation likelihood estimation (ALE) meta-analysis concerning cerebral activations during the study by Yuan and Brown (Yuan and Brown, 2015). Specifically, a cluster of activation comprising left sensorimotor and associative areas was found in the left frontal and parietal lobules. The primary motor and premotor cortices are commonly activated during motor control of the right hand, including handwriting. In particular, there was a significant activation in the location of Exner’s area, one of the brain regions identified by former studies on handwriting as “writing center” (Exner, 1881; Longcamp et al., 2003; Matsuo et al., 2003; Sugihara et al., 2006; Roux et al., 2010; Purcell et al., 2011), although its specificity for writing has been under debate (Yuan and Brown, 2015). We also observed the activation of the left IPL, one of the associative areas involved in visuomotor coupling and identified as the “parietal writing center” by Sugihara and colleagues (Sugihara et al., 2006). A large activation appeared in the right cerebellum, which is commonly considered part of the basic motoric component of writing (Yuan and Brown, 2015). Another cluster of activation consisted of regions ranging from the right SPL to the IPL, including the intraparietal sulcus already shown to be activated during handwriting (Yuan and Brown, 2015). Differently from Yuan and Brown, in our work, activation also appeared in the left middle temporal gyrus and the left middle occipital gyrus, areas situated in proximity of the visual word form area as identified by Nakamura et al. (2012).

The brain activation pattern of PwMS was reduced as compared with that of HC, as pointed out by the lack of significant activation clusters in the contrast analysis PwMS > HC and mainly referred to the left motor areas and the right cerebellum. In addition, activation of the right SPL was observed. Notably, these areas are a subgroup of those observed in HC, showing that, in this group of PwMS, there were no compensatory areas significantly active during the handwriting task. Also, this finding indicates that the activities of these areas were sufficient to allow PwMS to achieve the task, although characterized by larger duration in its accomplishment as compared with that of HC.

In the present study, we assessed whether handwriting spontaneous tempo could be associated with the activity of the brain areas, which resulted in statistical significance from the contrast analysis HC > PwMS (the left insula, caudate and fusiform gyrus, right middle occipital gyrus and IPL, bilateral cerebellar lobules IV–VI, and thalamus). In detail, we investigated how activity changes in these areas were related to the duration of execution of the sentence, words, and duration of the spacing between words. In particular, the right IPL negatively correlated with the duration of the sentence. The right IPL was previously shown to be activated during handwriting (Yuan and Brown, 2015); however, a clear explanation of its role in this task was not given.

Several models of IPL function have been proposed (Husain and Nachev, 2007). In Goodale and Milner’s view, the dorsal stream (or “vision-for-action” pathway) delivers information directly to the motor system for immediate use for reaching, grasping, or eye movements, whereas the ventral stream is considered as the “vision-for-perception” pathway, mainly for recognition and discrimination of visual shapes and objects, but it might have a role in movement planning based on memory of the object and its relationship to other items (Milner and Goodale, 2008).

On the other hand, Rizzolatti and Matelli suggested to consider the superior and inferior parts of the posterior parietal cortex (PPC) as belonging to two different streams: the SPL to a “dorso-dorsal” system dedicated to the online control of action and the IPL to a “ventrodorsal” stream essential for action understanding and spatial perception (Rizzolatti and Matelli, 2003). Specifically, these functions, together with peripersonal space representation, are represented by areas of the IPL where visual information from both the dorsal and ventral streams is integrated with motor information (Fogassi and Luppino, 2005).

The existence of segregated distinct brain networks including different portions of the PPC and carrying out different attentional functions was proposed by Corbetta and Shulman (Corbetta and Shulman, 2002). They argued that the SPL and parts of the intraparietal sulcus have a role in directing visual attention “top-down” toward locations or objects in the scene and in selecting responses of effectors (eye or limb). By contrast, the right temporo-parietal junction, a more ventral region in the PPC, acts as a “circuit breaker” for the dorsal system, directing attention to salient events.

Furthermore, concerning the non-spatial functions of the right IPL, Husain and Natchev observed that parts of the human IPL seem to be neither “dorsal” nor “ventral” (Husain and Nachev, 2007). They have non-spatial functions that are not related to object processing, as found in “ventral” stream temporal cortical areas. Instead, they could have a role in detecting salient new items embedded in a sequence of events and maintaining or controlling attention over time.

The right IPL was also proposed to be part of the “when” pathway, i.e., the brain circuit involved in processing the time in which visual events occur (Battelli et al., 2007). Indeed, studies involving healthy and cerebrally lesioned subjects demonstrated that the parietal lobe is involved in the analysis of time as well as space, for both visual and auditory stimuli (Husain and Rorden, 2003). In this context, studies on patients with lesions in the right IPL suggested a specific role for this brain area in perceptual abilities requiring the analysis of time (Husain et al., 1997; Battelli et al., 2001).

Here, we showed that the activity of the right IPL, and specifically BA40, is associated with handwriting spontaneous tempo. However, we cannot disentangle if the timing information is locally computed in a task-dependent manner or if the activity of these parietal neurons only reflects decision processes where the timing information might be computed upstream and then transmitted to parietal neurons associated with specific response systems (Ivry and Spencer, 2004). In our work, subjects were asked to write the sentence at their spontaneous tempo, which can be influenced by cerebellum and basal ganglia activities (Schwartze et al., 2011, 2016). Interestingly, in a recent resting-state fMRI study, it has been demonstrated that the anterior IPL, mainly corresponding to BA40, has a functional connectivity with the basal ganglia structures and the cerebellum greater than that observed for the posterior IPL (Zhang and Li, 2014). Therefore, one could assume that timing information is processed in subcortical structures, such as basal ganglia and cerebellum, and right IPL integrates timing and handwriting task, with visuomotor information playing a role in the attention-dependent temporal processing.

## Conclusion

Following all these findings, it comes out that although one of the main roles of PPC is to integrate sensory and motor signals in order to accomplish sensorimotor transformations necessary for motor planning and sensory guidance of movements, PPC, and in particular the right IPL, is also involved in higher-order aspects of motor control linking action to time perception and attention.

Here, we propose that the right IPL might be considered a “writing tempo center,” which detects salient events embedded in a sequence of events and controls attention, taking into account the time in which visual events occur and thus monitoring the temporal components of handwriting.

## Data Availability Statement

The raw data supporting the conclusions of this article will be made available by the authors, without undue reservation.

## Ethics Statement

The studies involving human participants were reviewed and approved by Comitato Etico Regionale Liguria, IRCCS Azienda Ospedaliera Universitaria San Martino—IST, Genoa, Italy; P.R. 258REG2015. The patients/participants provided their written informed consent to participate in this study.

## Author Contributions

LB, AB, and MB conceived, designed the experiment, and drafted the manuscript. LB, AB, and LP acquired data. LB and AB analyzed the data. All authors interpreted data and approved the final version of the manuscript. GB revised the manuscript. All authors contributed to the article and approved the submitted version.

## Conflict of Interest

The authors declare that the research was conducted in the absence of any commercial or financial relationships that could be construed as a potential conflict of interest.

## Abbreviations

HC: healthy controls
IPL: inferior parietal lobule
PwMS: people with multiple sclerosis
PPC: posterior parietal cortex
SPL: superior parietal lobule.
