# Supplementary material for: Right Inferior Parietal Lobule Activity Is Associated With Handwriting Spontaneous Tempo
Source: Front Neurosci. 2021 Jun 9;15:656856. doi: 10.3389/fnins.2021.656856 (PMC8219918; doi:10.3389/fnins.2021.656856)
Supplement: Supplementary file 1 [file Table_1.docx]

**Supplementary Material**

**Figures**

**Figure 1S**. (A) Experimental set-up and (B) definition of the kinematic parameters.

**
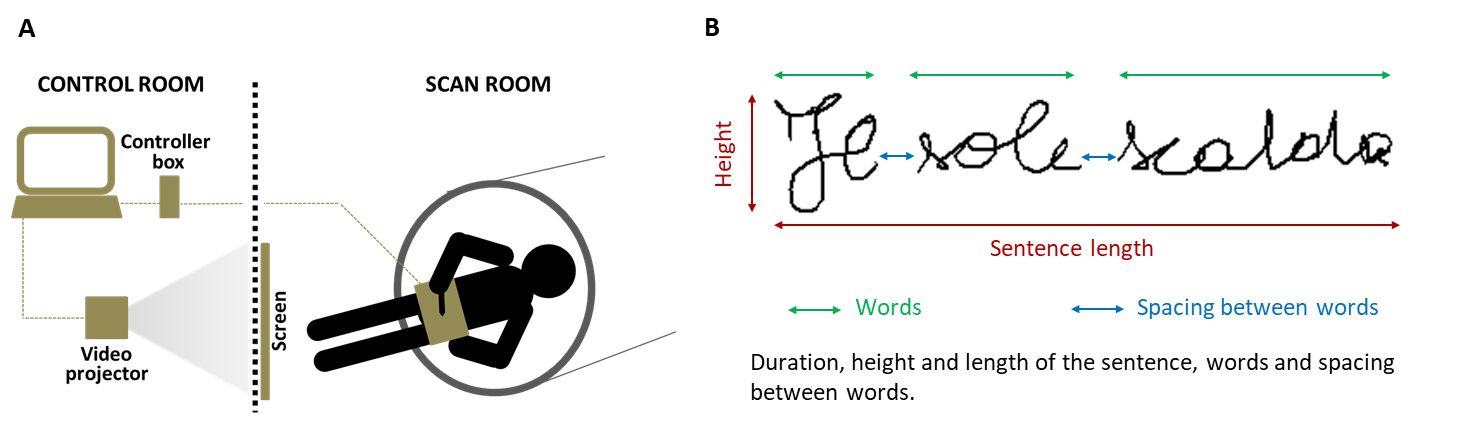
**

**Tables**

**Table 1S** Demographic and clinical characteristics of the people with multiple sclerosis (PwMS) included in the study. RR = relapsing–remitting; SP = secondary progressive.

| **Age (years)** | **Gender** | **MS phenotype** | **EDSS** | **Disease duration (years)** | **Disease-modifying therapy** | **Affected upper limb** |
| --- | --- | --- | --- | --- | --- | --- |
| 53 | F | RR | 1.5 | 5 | --- | Left |
| 32 | F | RR | 4 | 8 | Fingolimod | Left |
| 62 | M | SP | 6 | 41 | Methotrexate | Bilateral |
| 44 | F | RR | 2 | 19 | Fingolimod | Right |
| 43 | F | RR | 6 | 23 | Interferon | Right |
| 57 | M | SP | 6 | 19 | Interferon | Right |
| 53 | M | RR | 5 | 13 | --- | Right |
| 31 | F | RR | 5 | 8 | Natalizumab | Bilateral |
| 47 | F | RR | 2 | 10 | Interferon | Bilateral |
| 62 | M | RR | 5 | 6 | Fingolimod | Left |
| 55 | F | RR | 4 | 18 | Interferon | Bilateral |
| 36 | F | RR | 2 | 9 | --- | Left |
| 43 | F | SP | 2 | 25 | Fingolimod | Bilateral |
| 40 | F | RR | 2 | 10 | Fingolimod | Right |
| 52 | M | SP | 6 | 32 | --- | Left |
| 27 | M | RR | 2 | 5 | --- | Bilateral |
| 46 | F | RR | 4 | 22 | Fingolimod | Bilateral |
| 29 | F | RR | 1 | 8 | Fingolimod | Bilateral |

**Table 2S**. Brain regions significantly activated during the handwriting motor task in the two groups (p<0.05 FWE-corrected, minimum cluster size k = 20 voxels).

| **Group** | **Cluster Size** | **Voxel T** | **Voxel Z** | **MNI Coordinate: x y z (mm)** | **Laterality** | **Anatomical Location** | **Brodmann's Area** |
| --- | --- | --- | --- | --- | --- | --- | --- |
| **HC** | 4672 | 14.36 | Inf | -28 -22 54 | Left | Precentral Gyrus | 6 |
|  |  | 12.05 | 7.47 | -38 -14 54 | Left | Precentral Gyrus | 4 |
|  |  | 10.25 | 6.88 | -30 -52 58 | Left | Inferior Parietal Lobule | 40 |
|  | 987 | 8.87 | 6.34 | 2 -64 -22 | Right | Cerebellum (lobule VI) |  |
|  |  | 8.46 | 6.17 | 10 -56 -12 | Right | Cerebellum (lobules IV-V) |  |
|  |  | 8.36 | 6.12 | 6 -70 -14 | Right | Cerebellum (lobule VI) |  |
|  | 739 | 7.77 | 5.85 | 44 -34 46 | Right | Inferior Parietal Lobule | 40 |
|  |  | 7.33 | 5.64 | 32 -48 54 | Right | Precuneus | 7 |
|  |  | 7.32 | 5.63 | 22 -58 54 | Right | Precuneus | 7 |
|  | 34 | 7.01 | 5.48 | -14 -18 0 | Left | Thalamus |  |
|  | 74 | 6.82 | 5.38 | -42 -64 -6 | Left | Middle Temporal Gyrus | 37 |
|  |  | 5.78 | 4.79 | -46 -72 2 | Left | Middle Occipital Gyrus | 37 |
|  | 64 | 6.49 | 5.2 | 30 -72 30 | Right | Precuneus | 19 |
|  |  | 6.37 | 5.13 | 26 -68 40 | Right | Superior Parietal Lobule | 7 |
|  | 29 | 5.9 | 4.86 | 44 -60 -10 | Right | Inferior Temporal Gyrus | 19 |
| **PwMS** | 2144 | 10.93 | 7.11 | -28 -22 54 | Left | Precentral Gyrus | 6 |
|  |  | 9.79 | 6.71 | -24 -14 62 | Left | Middle Frontal Gyrus | 6 |
|  |  | 9.36 | 6.54 | -38 -14 54 | Left | Precentral Gyrus | 4 |
|  | 24 | 7.52 | 5.73 | -10 -6 72 | Left | Superior Frontal Gyrus | 6 |
|  | 48 | 7.43 | 5.69 | 4 -54 -16 | Right | Cerebellum (lobules IV-V) |  |
|  | 22 | 6.43 | 5.17 | 16 -70 58 | Right | Superior Parietal Lobule | 7 |
